# Virus Prevalence and Genetic Diversity Across a Wild Bumblebee Community

**DOI:** 10.3389/fmicb.2021.650747

**Published:** 2021-04-22

**Authors:** David J. Pascall, Matthew C. Tinsley, Bethany L. Clark, Darren J. Obbard, Lena Wilfert

**Affiliations:** ^1^Institute of Biodiversity, Animal Health and Comparative Medicine, Boyd Orr Centre for Population and Ecosystem Health, University of Glasgow, Glasgow, United Kingdom; ^2^Centre for Ecology and Conservation, University of Exeter, Cornwall, United Kingdom; ^3^Biological and Environmental Sciences, University of Stirling, Stirling, United Kingdom; ^4^BirdLife International, The David Attenborough Building, Cambridge, United Kingdom; ^5^Environment and Sustainability Institute, University of Exeter, Cornwall, United Kingdom; ^6^Institute of Evolutionary Biology, University of Edinburgh, Edinburgh, United Kingdom; ^7^Institute of Evolutionary Ecology and Conservation Genomics, Ulm University, Ulm, Germany

**Keywords:** virus community ecology, disease ecology, bumblebees (Bombus), virus diversity, pollinators, Wildlife epidemiology

## Abstract

Viruses are key population regulators, but we have limited knowledge of the diversity and ecology of viruses. This is even the case in wild host populations that provide ecosystem services, where small fitness effects may have major ecological impacts in aggregate. One such group of hosts are the bumblebees, which have a major role in the pollination of food crops and have suffered population declines and range contractions in recent decades. In this study, we investigate the diversity of four recently discovered bumblebee viruses (Mayfield virus 1, Mayfield virus 2, River Liunaeg virus, and Loch Morlich virus), and two previously known viruses that infect both wild bumblebees and managed honeybees (Acute bee paralysis virus and Slow bee paralysis virus) from isolates in Scotland. We investigate the ecological and environmental factors that determine viral presence and absence. We show that the recently discovered bumblebee viruses were more genetically diverse than the viruses shared with honeybees. Coinfection is potentially important in shaping prevalence: we found a strong positive association between River Liunaeg virus and Loch Morlich virus presence after controlling for host species, location and other relevant ecological variables. We tested for a relationship between environmental variables (temperature, UV radiation, wind speed, and prevalence), but as we had few sampling sites, and thus low power for site-level analyses, we could not conclude anything regarding these variables. We also describe the relationship between the bumblebee communities at our sampling sites. This study represents a first step in the description of predictors of bumblebee infection in the wild.

## Introduction

Viruses are among the most abundant and diverse groups of organisms on Earth and have a key role in regulating natural populations (Wommack et al., 2015); wherever they are looked for, they are found as obligate pathogens. Despite this, viral ecology in natural populations remains understudied. The development of relatively cheap and easily applied molecular techniques has allowed the detection and identification of potentially pathogenic organisms within both the host and the environment, enabling the systematic study of viral ecology in wild populations [e.g., Webster et al. (2015)]. This is especially important for threatened host species, where understanding the viral burden may have conservation implications (Gordon et al., 2015).

Pollinators are economically important and threatened, and, as such, an understanding of their viruses is important. Over 50 viruses have now been described in bees, and their importance to survival is well recognized [e.g., McMahon et al. (2018)]. However, the majority of this work has been performed in the European honeybee, *Apis mellifera*, thus the knowledge of the ecology and evolution of viruses of bumblebees is more limited. Some of this work is transferable to bumblebees; for instance, viruses known from honeybees have pathogenic effects in the buff-tailed bumblebee, *Bombus terrestris* (Fürst et al., 2014; Graystock et al., 2016; Manley et al., 2017), and the prevalences of well-known honeybee viruses have been assayed across the United Kingdom (Fürst et al., 2014; McMahon et al., 2015). However, few predictors of viral infection in bumblebees other than the presence of sympatric honeybees have been described in any depth, and little work has been performed characterizing the genetic diversity of most bee viruses.

Given the complexity of the pollinator system, it is unclear how much viral genetic diversity is expected in pollinator communities. Some viruses, such as the re-emerging Deformed wing virus, are more frequent in honeybee populations and infections in wild pollinator populations appear to be seeded from the honeybee reservoir (Fürst et al., 2014; Wilfert et al., 2016; Manley et al., 2019). The genetic diversity in wild pollinators for these viruses will likely represent a potentially non-random sample of viral diversity in its maintenance host. For viruses that are maintained in bumblebees, however, we expect diversity to be impacted by the normal evolutionary patterns of mutation, selection and drift. RNA viruses tend to experience relatively high mutation rates on the order of 10^–4^–10^–6^ subs/site/cell (Lauring and Andino, 2010; Sanjuán and Domingo-Calap, 2019), which can lead to the generation of large amounts of viral diversity within a host, sometimes termed a quasispecies [reviewed in Lauring (2020)]. This is due to a variety of factors. Often RNA-dependent RNA-polymerases lack proofreading through 3′-exonuclease activity, which makes them error-prone, as genome copying mistakes are less likely to be corrected (Smith, 2017). Additionally, interactions with host proteins can cause extra mutations above those induced by the RNA-dependent RNA-polymerase (Sanjuán and Domingo-Calap, 2019). The realized diversity is then determined by the fate of these mutations. This depends on the efficiency of selection on those with selective effect, and the effective population size for those with no impact on fitness, or an impact on fitness so small as to be invisible to selection. Depending on the population size and the historical selection regime, this could lead to very high diversity. For viruses maintained in bumblebees, low genetic diversity would be an indication of a major selective event or recent bottleneck. If viruses circulate freely within the bumblebee community, no differences in the pattern of genetic diversity would be expected. If, however, certain species would show lower infection prevalences, due to e.g., resistance mutations or a stronger immune system due to ecological conditions favoring certain species, genetic diversity would be expected to be reduced if these species do not sample viral genetic diversity randomly.

Co-occurrences of particular pathogens have been observed in many species and can drive infection dynamics with certain combinations being over- or under-represented relative to chance expectations (Johnson et al., 2015; Tollenaere et al., 2016). This may be due to synergistic or antagonistic interactions between pathogens: for example, synergistic effects can occur if damage to tissues caused by a primary infection allows easier access for secondary pathogens (Joseph et al., 2013); antagonistic effects can, for example, occur due to competitive exclusion of pathogens with the same niche within the host (Amaku et al., 2013). Additionally, if the pathogens under study have differing annual periodicity in prevalence, as is observed in multiple bee viruses (Glenny et al., 2017; Faurot-Daniels et al., 2020), then there will be a changing co-occurrence between them expected by chance throughout the year, making the detection of deviations from the underlying expected pattern more difficult to detect from data with varying sampling dates. Very few studies in pollinators have looked for these between pathogen interactions in a statistically rigorous manner.

Differences in viral prevalence between hosts or locations at a given time can be explained by a variety of environmental and biotic factors. For pollinators, these can include the presence of host species like managed honeybees and their viral vector *Varroa destructor* (Fürst et al., 2014; Manley et al., 2019, 2020) or other dominant bee species (Graystock et al., 2020), all of which modify exposure to viruses, as well as landscape effects such as the intensity of agricultural land-use and habitat loss (Figueroa et al., 2020; Daughenbaugh et al., 2021) or the presence of particular floral resources (Adler et al., 2020). Here, we focus on under-explored abiotic factors, which can be important if viruses are spread by environmental contamination or aerosolisation, then abiotic factors can be important. In bumblebees, infection is often thought to take place at flowers (Durrer and Schmid-Hempel, 1994; McArt et al., 2014; Graystock et al., 2015; Agler et al., 2019) and so factors that reduce contamination of floral structures may be predicted to reduce the rate of infection in the general bumblebee population (Adler et al., 2020); obvious mechanisms are viral deactivation, flower visitation rates and physical cleaning. The rate of viral deactivation can be increased in high temperatures, both independently and through an interaction with relative humidity (Mbithi et al., 1991) and high UV levels may deactivate virus particles rapidly (Lytle and Sagripanti, 2005). Bees must physically reach the flowers where infection can occur, so factors that change the rate of contact of workers with heavily contaminated flowers may also modify viral prevalence. Wind speed affects the relative rates of pollen and nectar collection (Peat and Goulson, 2005), which may alter flower visitation and the energetic costs of foraging (Wolf et al., 1999), consequently affecting susceptibility to infection. Precipitation can also limit bumblebee foraging and therefore flower contamination risk (Peat and Goulson, 2005). Finally, heavy rain and strong winds may physically clean the flowers. However, it could also be envisioned to impact infection risk through changes in intra-colony contact rates. Environmental conditions would only be expected to lead to interspecific prevalence differences locally through species-specific effects on bee behavior.

Here, we present an exploratory investigation into the determinants of viral prevalence and genetic diversity in wild bumblebee populations consisting of 13 species from nine sites across Scotland. We consider the genetic diversity of four recently discovered bumblebee viruses Mayfield virus 1 (MV1), Mayfield virus 2 (MV2), River Liunaeg virus (RLV), and Loch Morlich virus (LMV), where fitness effects have not yet been tested, and contrast this with two viruses known from honeybees, Acute bee paralysis virus (ABPV), and Slow bee paralysis virus (SBPV). We also explore whether or not there are facilitative or suppressive interactions between these viruses and explore the effect of differences in temperature, UV radiation, wind speed and precipitation on their prevalences. We show that the viruses described only in bumblebees are universally more diverse than ABPV and SBPV and that there is a strong positive association between LMV and RLV infection.

## Materials and Methods

### Sampling Regime and Molecular Work

**FIGURE 1:**
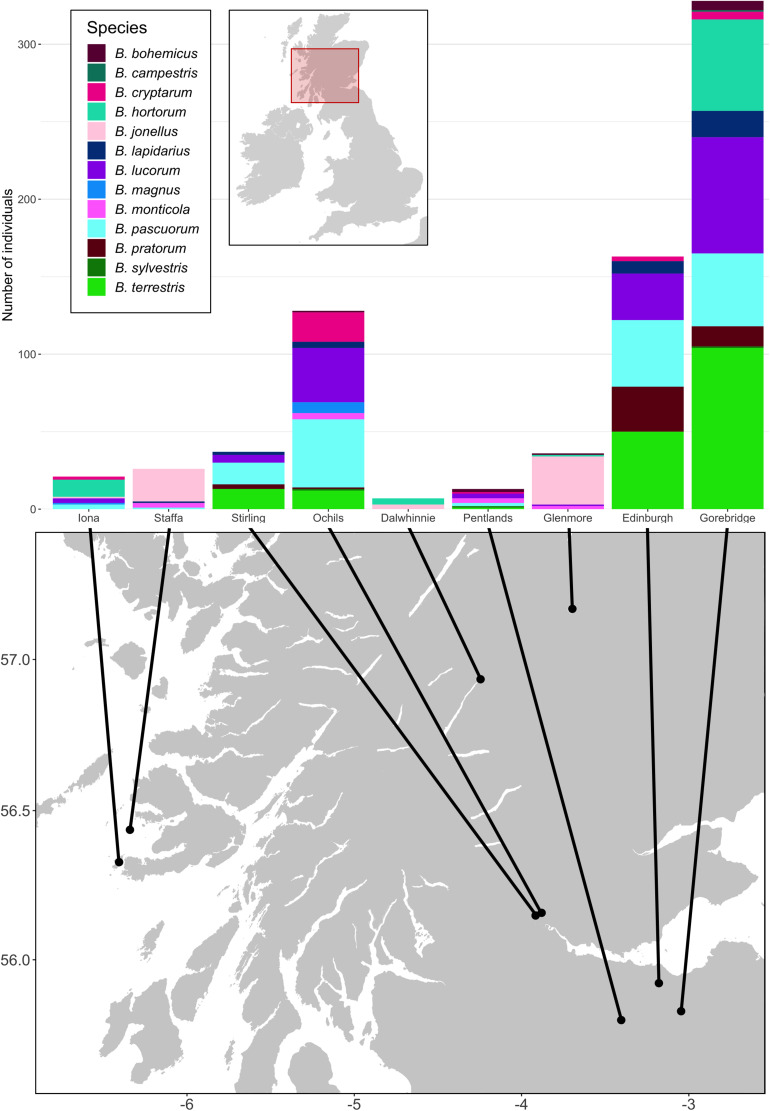
The locations of the sampling sites and species distributions and sample sizes of the bumblebees caught at them. For each site, all bees taken from that site were sampled on the same day. The Ochil Hills, Glenmore, Dalwhinnie, Stirling, Iona, Staffa, and the Pentlands were sampled in 2009, while Edinburgh and Gorebridge were sampled in 2011. Map adapted from tiles by Stamen Design, under Creative Commons (CC BY 3.0) using data by OpenStreetMap, under the Open Database License.

Samples were derived from the field collections described in Pascall et al. (2018). Briefly, we collected a total of 759 bumblebees of 13 species from nine sites across Scotland, United Kingdom (Supplementary Table 1; Figure 1). The Ochil Hills, Glenmore, Dalwhinnie, Stirling, Iona, Staffa, and the Pentlands were sampled in 2009, while Edinburgh and Gorebridge were sampled in 2011 (see Supplementary Table 2 for exact sampling dates). On Iona and Staffa, 59 *Bombus muscorum* were caught, but did not go forward to the extraction stage and were instead used in Whitehorn et al. (2011). We performed individual RNA extractions using TRIzol (Life Technologies) following the manufacturers’ standard protocol. RNA was transcribed into cDNA using random hexamers and goScript MMLV reverse transcriptase (Promega) following the manufacturers’ instructions.

In this study, we assayed the prevalence of Mayfield virus 1 (MV1), Mayfield virus 2 (MV2), River Luinaeg virus (RLV), Loch Morlich virus (LMV) at the individual level by RTPCR (Supplementary Table 3). We tested a subset of the samples for Slow bee paralysis virus (SBPV) (*n* = 544) and Acute bee paralysis virus (ABPV) (*n* = 385); these were the only common honeybee viruses detected by next generation sequencing analyses (Pascall et al., 2018).

### Diversity Analysis

To analyse sequence diversity, we used the raw reads from the RNA sequencing described in Pascall et al. (2018). For context, the mapping statistics as calculated for each virus in Pascall et al. (2018) are provided in Supplementary Table 4. Briefly, these consist of 100 bp-paired end RNA-Seq data from pools of *B. terrestris* (239 individuals), *Bombus pascuorum* (212 individuals) and *Bombus lucorum* (182 individuals), each sequenced twice by BGI Genomics, once using duplex specific normalization and once using poly-A selection both of which reduced the contribution of ribosomal RNAs to the final sequencing data, and a pool of mixed *Bombus* species (293 individuals), sequenced only with poly-A selection. MV1, MV2, RLV, LMV, SBPV Rothamsted (EU035616.1) and ABPV (AF486072.2) sequences were taken from GenBank and aligned on the TranslatorX server (Abascal et al., 2010), using its MAFFT setting (Katoh and Standley, 2013). Post-alignment, we manually trimmed sequences to the conserved region of the RdRp gene, minus eight codons, owing to the shortness of the RLV sequence. Trailing regions of 200 base pairs at both ends were retained so that reads were not prevented from mapping due to an overhang. This gave final sequence lengths of 1,483, 1,483, 1,536, 1,501, 1,519, and 1,522 base pairs for MV1, MV2, RLV, LMV, SBPV, and ABPV, respectively, to use as mapping references. Raw bioinformatic reads were trimmed in sickle version 1.33 using the default parameters (Joshi and Fass, 2011). Overlapping mate reads were combined by FLASH version 1.2.11 using the default settings (Magoč and Salzberg, 2011). Reads were aligned to the RdRp sequences generated above using MOSAIK version 2.1.73 (Lee et al., 2014). Both merged reads and singletons from the sickle run were aligned together in the single end setting. Unmerged paired end reads were separately aligned using the paired end setting. In both cases, a quality threshold of 30 was used to remove ambiguously mapping reads. SAM files were recombined after the fact using SAMtools version 1.5 (Li et al., 2009). There was high coverage of SBPV, MV1, and MV2, so duplicate sequences were not marked, as per best practice (McKenna et al., 2010). Variants were called using the default settings in LoFreq^∗^ version 1.2.1 (Wilm et al., 2012). Base quality scores were recalibrated using the outputted vcf file in GATK (DePristo et al., 2011). Variant calling and recalibration were repeatedly performed until the base quality scores converged to a stable distribution (a total of four recalibrations). Once the score distribution stabilized, variant calling was performed to generate a set of variants for the entire sample. These variants were used to recalibrate the scores of each species-specific mapping and generate species level variant calls. If the median depth over called differences from the consensus was less than 20, species-virus combinations were removed from the variant analysis. *B. lucorum* was analyzed for SBPV (median depth: 521 reads), ABPV (median depth: 189 reads) and MV1 (median depth: 5373.5 reads). *B. terrestris* was analyzed for SBPV (median depth: 400 reads), MV1 (median depth: 5786.5 reads) and MV2 (median depth: 35 reads). *B. pascuorum* was analyzed for ABPV (median depth: 2,180 reads), SBPV (median depth: 69,282 reads), and MV2 (median depth: 5433.5 reads). The mixed *Bombus* pool was analyzed for all six viruses (ABPV median depth: 162 reads; SBPV median depth: 77,888 reads; RLV median depth: 31 reads; LMV median depth: 26 reads; MV1 median depth: 25.5 reads; MV2 median depth: 1,410 reads).

The number of polymorphic sites was calculated for each virus. Variants with allele frequencies of 1 were removed as these represent fixed differences from the underlying reference sequence. To measure genetic diversity, we used these counts to approximate Watterson’s estimator (Watterson, 1975) for each host-virus combination. We had to account for the fact that the make-up of the pools was not precisely the same as the samples that were tested by PCR. As such, we predicted the status of each of the untested individuals in the pools from the model discussed above and took the median and a 90% central credible interval of the number of additional positives (over those confirmed by PCR). We then used these numbers to give bounds on the approximation to Watterson’s estimator, by looking at the value of the estimator at those three estimates of the number of infected individuals. The equation used was:

$$\theta_{a\,p\,p\,r\,o\,x}=\frac{n}{l\,\sum_{i=1}^{p}\frac{1}{i}}$$

where θ_approx_ is the approximation to Watterson’s estimator, *n* is the number of variants, *l* is the length of the sequence and *p* is the number of putative positives. This method makes two strong assumptions: (1) there is no mixed infection of viral variants in individuals (i.e., that one extra individual represents a single extra count for the harmonic partial sum in the denominator of Watterson’s estimator) and (2) all variants present are detectable. The impact of deviation from the first assumption is likely to be small. The marginal change in the partial sum in the denominator decreases with every extra count, so a few missed counts will result in little change to the resultant estimate. The second assumption is more influential, given the larger impact that a missing variant has on the generated number. Given this, we acknowledge that our presented estimates may be conservative. We tested for an association between the approximation to Watterson’s estimator and the median read depth over called variants using Kendall’s tau, a method of rank-based correlation that does not make assumptions about the normality of the two variables. A 95% confidence interval was generated for the correlation using a naïve percentile bootstrap.

### Prevalence and Climatic Association

Climatic data for each of the nine sites at which bees were collected was taken from the WorldClim database at 1 km resolution (Fick and Hijmans, 2017). Predictions for July and August derived from data from 1960 to 2010 were extracted for mean daily maximum temperature, mean precipitation, mean solar radiation and mean wind speed at the grid reference for the sites with a buffer area of 2 km to account for the fact that bumblebees forage over approximately that distance (Osborne et al., 2008; Wolf and Moritz, 2008). All values were averaged to generate a consensus value for that site and then mean-centered and scaled to unit standard deviation. At this point, we tested the correlation between the variables. Mean solar radiation and mean daily maximum temperature were highly correlated (Pearson correlation: 0.78), so only mean maximum temperature was carried forward. All remaining variables had low Pearson correlations between them in the range of −0.4 to 0.4.

We tested associations between individual prevalence and climate data using Stan version 2.18.2 (Carpenter et al., 2017) via the RStan interface (Stan Development Team, 2016) in R version 3.6 (R Core Development Team, 2016). A multivariate probit model was fitted, with random host, location and host-location effects, and mean maximum temperature, precipitation, and wind speed as fixed effects for each virus. The usage of the multivariate probit allows us to test for excess co-infection between viruses in the study, i.e., coinfection beyond random expectations after the effect of shared covariates has been removed. As the number of sampling locations was small, we expected our ability to accurately determine the size and direction of effects caused by ecological covariates would be limited. To reduce the effect of drawing spurious conclusions due to our small number of sites, we applied regularization as recommended by Lemoine et al. (2016), using a regularizing prior distribution. The global intercept for each virus was given a Gaussian (mu = 0, sigma = 10) prior, which does not substantially penalize low probabilities. Each fixed effect coefficient was given a Gaussian (mu = 0, sigma = 1) prior, which, given that the fixed effects act at the site level, should dominate the likelihood if the effect is small. Random effects were drawn from normal distributions centered at 0 with estimated standard deviations. In all cases, the standard deviations were given Exponential (lambda = 2) hyperpriors, which are only weakly informative on the logit scale when the data is informative for the standard deviation. The correlation in residuals for the multivariate normal was given a near flat prior using an LJK (eta = 1) prior. While the Stan code used can regularly give outputs with divergent transitions, the presented model had no divergent transitions over 24,000 samples, tail and bulk effective sample sizes of over 400 for all parameters and no Bayesian fraction of missing information warning. We did not perform model selection given our regularizing priors, and statements are made based on estimates from the full model.

### Community Similarity

To estimate host community similarity between sampling sites, we estimated the underlying sampling probability of each bumblebee species at each site by treating the observed samples as being drawn from a multinomial distribution with 24 categories, corresponding to the 24 bumblebee species in the United Kingdom. This analysis included the *Bombus muscorum* samples that were excluded from the other analyses, so as not to bias the sampling results. We use a Dirichlet prior with these 24 categories and a concentration parameter of 1 for each category, implying complete uncertainty about the underlying probability. This has the advantage that the posterior has a known analytical form. Probabilities were estimated independently for each site. Ten thousand simulations were taken from the posterior distributions generated for each site to generate possible values of the underlying sampling probabilities of each bumblebee species at each site, which we assume to be roughly equivalent to the frequency of that bumblebee species at that site. For each of the 10,000 simulations from the posteriors at the sites, we generated estimates of the community dissimilarity using the Morisita-Horn index (Horn, 1966), implemented in the R package vegan (Oksanen et al., 2017). We reported the posterior mode and 90% shortest probability intervals for the dissimilarity index.

## Results

### Diversity

**FIGURE 2:**
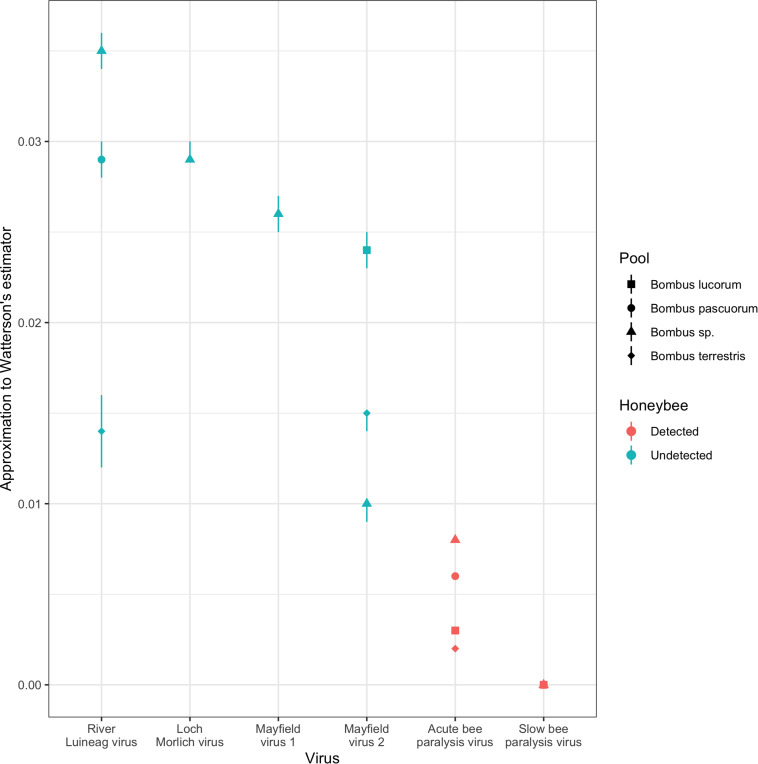
The diversity of Acute bee paralysis virus, Loch Morlich virus, Mayfield virus 1, Mayfield virus 2, River Luinaeg virus and Slow bee paralysis virus in a series of metatranscriptomic pools as measured by an approximation to Watterson’s estimator (see section “Materials and Methods”). The viruses are ordered from highest to lowest diversity in the mixed *Bombus* pool, the only pool for which all combinations were assayed. Errors correspond to estimation at the end points 90% credible interval for the number of extra untested positives from the pools (see section “Materials and Methods”). Combinations were excluded if the median read depth over called differences from the consensus was less than 20.

Over homologous genomic regions within the RdRp gene, there were large differences between viruses in genetic diversity as measured by our approximation to Watterson’s estimator (Figure 2; Supplementary Table 5). RLV, LMV, MV1, and MV2 all exhibited more diversity than SBPV and ABPV, with SPBV itself being considerably more diverse than ABPV. This is despite SBPV and ABPV being considerably more prevalent viruses than the other four. There was no detectable relationship between the read depth and the approximation to Watterson’s estimator (Kendall’s tau: 0.01, 95% CI: −0.31, 0.38; Supplementary Figure 1). The same genotypes of MV1 and MV2 are observed in both 2009 when Dalwhinnie, the Ochils and Iona were sampled and 2011 when Edinburgh, Gorebridge and the Pentlands were sampled, implying that the variants present in an area are stable over short periods. Additionally, as would be expected, most variation was in 3rd codon positions leading to either no amino acid replacements or replacements with similarly charged amino acids, and thus unlikely to affect protein function.

### Prevalence

**FIGURE 3:**
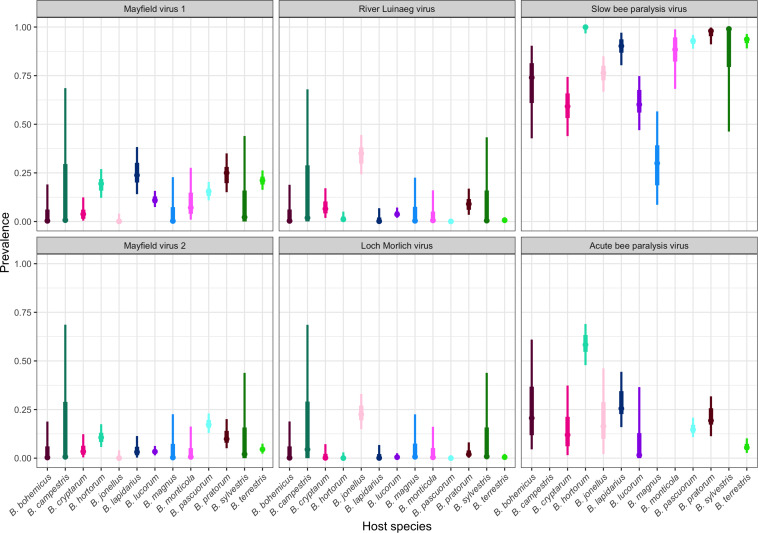
The prevalence of Acute bee paralysis virus, Loch Morlich virus, Mayfield virus 1, Mayfield virus 2, River Luinaeg virus, and Slow bee paralysis virus in each sampled host species. The point estimate is the posterior mode, with 50% shortest posterior intervals represented by the thick lines and 90% shortest posterior intervals represented by the thin lines. Shortest posterior intervals are a continuous credible interval (measured on the scale of the parameter) that contains 90% of the posterior density, that is, an interval that has a 90% chance of containing the correct parameter value under the model, the data and the priors. Untested combinations are left blank. Species are colored by their corresponding color in Figure 1 for ease of reading.

There were large differences in the prevalences of the viruses, all of which are +ssRNA picorna-like viruses, between sites (Figure 3, all host-location combinations in Supplementary Figure 2). When broken down to the specific host-location level, sample sizes for many species become small, so the uncertainty around the modal prevalences is correspondingly large.

River Luinaeg virus (RLV) was detected in *B. jonellus* at all sites where the species was sampled, with prevalences of approximately 25% or higher detected at multiple sites. The prevalence was similarly high in *Bombus pratorum*. Intermediate prevalences were detected in *Bombus cryptarum*. Low levels of infection with RLV were detected in *B. lucorum* with the prevalences of the virus appearing to be considerably higher in this species in Stirling and the Pentlands. Loch Morlich virus (LMV) appears to exhibit much higher species specificity with 13/16 detections being in *B. jonellus*. It was also strongly associated with RLV, with 13/16 detections being coinfections. No species other than *B. jonellus* were detected with LMV infection in the absence of RLV coinfection. Mayfield virus 1 (MV1) appears to be a generalist, with frequent infections across bumblebee species. Its prevalence data showed large differences between the degree of infection of different bumblebee species between sites. Edinburgh and Gorebridge, two sites around 15 km apart with large sample sizes, have dramatically different MV1 prevalences in *B. terrestris*, *B. pratorum*, and *B. pascuorum*, being between 30 and 60% in Edinburgh, and below 15% in all species in Gorebridge. Mayfield virus 2 (MV2) shows a similar pattern but without obvious differences in infection levels between sites. The prevalence of MV2 is generally lower than that of MV1, but beyond that, the range of species infected is largely similar. Acute bee paralysis virus (ABPV) was found at intermediate modal prevalences of above 10% in all species apart from *B. terrestris* and *B. lucorum*. The prevalence of SBPV was universally high.

### Coinfection

We also tested for excess co-infection beyond random between viruses using multivariate probit models that allowed us to calculate the correlation in the error terms of the multivariate normal latent variable. This measures the degree to which, after accounting for the predictors, there is still shared error, as caused by unobserved factors affecting infection risk. In this case, these measure the extent to which there is excess coinfection after accounting for the location of sampling, the species of bee and the various location-level environmental variables. Some viruses exhibited excess coinfection (Table 1). RLV and LMV showed a strong positive correlation (mean correlation: 0.73), consistent with the high levels of coinfection noted above; the error correlation between these two viruses was the only one where the bulk of the posterior was not close to zero. There was also some indication of a negative association between MV2 and SBPV, with the bulk of the posterior supporting a correlation of below zero, but the variance of the posterior was such that this cannot be stated with great certainty.

**TABLE 1:** The posterior correlations of the errors of each virus from the multivariate probit model, measuring the degree of co-occurrence.

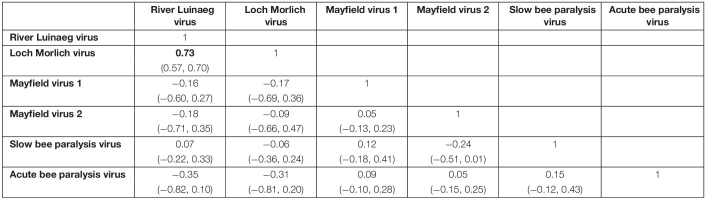

*Positive numbers represent excess co-occurrence beyond that predicted by covariates and negative numbers represent a dearth of double infections. The bolded observation shows the virus pair that has well supported excess co-occurrence after controlling for other effects. A 90% shortest posterior intervals for each correlation are shown in brackets.*

### Environmental Covariates

**FIGURE 4:**
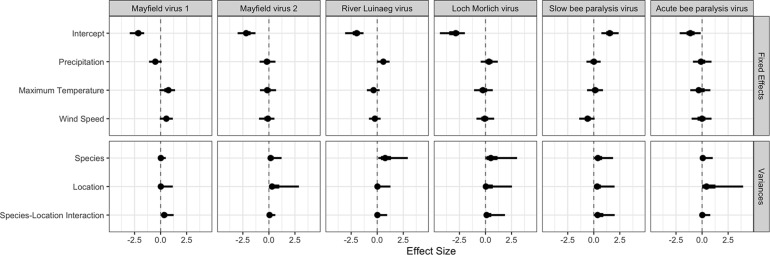
The estimates for each parameter in each virus from the multivariate probit model. The point estimate is the posterior mode, with 50% shortest posterior intervals represented by the thick lines and 90% shortest posterior intervals represented by the thin lines. Shortest posterior intervals are a continuous credible interval (measured on the scale of the parameter) that contains 90% of the posterior density, that is an interval which has a 90% chance of containing the correct parameter value under the model, the data and the priors.

Viral prevalence was related to environmental covariates in some cases (Table 2 and Figure 4, all parameters in Supplementary Figure 3). Higher levels of precipitation had a high posterior probability of being associated with higher prevalences of River Luinaeg virus (posterior probability: 95%). There was some evidence that more precipitation, higher maximum temperatures (which were highly correlated with solar radiation) and higher wind speeds were associated with higher prevalences of Mayfield virus 1 (posterior probability: 92, 94, and 93%, respectively). For most covariates, however, the bulk of the posterior distribution lay close to zero and did not shift considerably from the prior indicating a lack of between site resolution. Given the low site-level sample size, the effects described in this section may be noise.

**TABLE 2:** The posterior means and 90% shortest posterior intervals of the coefficients of the effect of each environmental covariate on each virus on the link scale from the multivariate probit model.

	**Precipitation**	**Maximum temperature**	**Wind speed**
**River Luinaeg virus**	0.60 (0.00, 1.19)	−0.36 (−0.99, 0.23)	−0.22 (−0.79, 0.33)
**Loch Morlich virus**	0.37 (−0.46, 1.18)	−0.18 (−1.01, 0.70)	−0.02 (−0.87, 0.83)
**Mayfield virus 1**	−0.50 (−1.11, 0.11)	0.66 (−0.11, 1.39)	0.56 (−0.08, 1.17)
**Mayfield virus 2**	−0.13 (−0.90, 0.63)	−0.10 (−0.86, 0.69)	−0.20 (−0.95, 0.53)
**SBPV**	−0.03 (−0.70, 0.66)	0.11 (−0.64, 0.88)	−0.64 (−1.40, 0.08)
**ABPV**	0.01 (−0.88, 0.91)	−0.15 (−1.13, 0.80)	−0.04 (−1.00, 0.91)

### Bumblebee Community Similarity

There were obvious differences in bumblebee community structure between our Scottish sampling sites. The locations we sampled in the south had *B. terrestris*, *B. pascuorum*, and *B. lucorum* dominated communities, whereas those further north had *Bombus jonellus* and *Bombus hortorum* dominated communities (Figure 1 and Table 3). The Pentlands, a range of hills in southern Scotland (Figure 1), appeared to represent a third type of community: the presence of *Bombus monticola*, otherwise only found in the highland sites, and an equivalent frequency of *B. pascuorum* and *B. lucorum* makes the community look like a blend of the other community types. This is potentially due to its higher elevation and habitat being similar to the north with large numbers of heathland plants while being situated in the south. The potential uniqueness of the Pentlands relative to other sampled locations, while it makes sense from an environmental perspective, should be considered tentative, given that the sample from that location was one of the smallest, of only 13 bees.

**TABLE 3:**
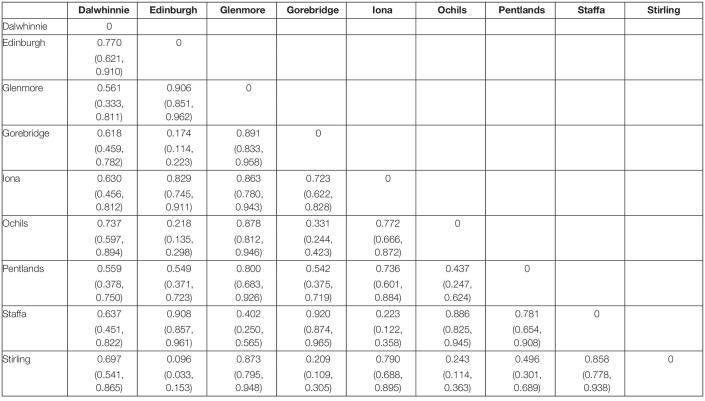
The Morisita-Horn dissimilarities of the bumblebee compositions of the different sampling sites.

## Discussion

In this study, we explored the genetic diversity and distribution of the viruses of wild bumblebees. We found that all the viruses detected only in bumblebees have considerably higher genetic diversity than the viruses shared with honeybees. Additionally, we found evidence of a positive association between River Luinaeg virus and Loch Morlich virus. We tested for an association between several environmental variables and prevalence, but due to low power, we cannot conclude anything on this topic. We also described the degree of dissimilarity of the bumblebee community compositions of our sampling sites.

### Diversity

Both Acute bee paralysis virus (ABPV) and Slow bee paralysis virus (SBPV) show considerably less diversity than Mayfield virus 1 (MV1), Mayfield virus 2 (MV2), River Luinaeg virus (RLV) and Loch Morlich virus (MLV) within the study region. ABPV and SBPV were initially described in honeybees (Bailey et al., 1963; Bailey and Gibbs, 1964), while the other four viruses were found in bumblebees and have not been recorded in honeybees at this point (Pascall et al., 2018). We cannot, however, rule out the possibility that there may be a large number of host species for these viruses that are not currently known, with this important caveat made, we will assume for the sake of discussion that MV1, MV2, RLV, and MLV are all bumblebee limited, given the lack of evidence to the contrary. Our approximation to Watterson’s estimator shows that the diversity in ABPV and SBPV remains low in bumblebees across the short period between 2009 and 2011. We do not have an explanation for this observation, but we discuss some possibilities below.

It is likely that much of the observed variation in the “bumblebee viruses” LMV, MV1, MV2, and RLV is neutral, as most variation is at 3rd codon positions and codes for either identical or similarly charged amino acids, which are unlikely to have large fitness effects in either direction on the virus. Given the frequent bottlenecking that occurs during transmission (Zwart and Elena, 2015), even mutations that cause phenotypic changes with a negative impact on fitness may be inefficiently selected against. It would be expected that given a constant mutation rate, the amount of diversity within a host would be related to the replication rate of the virus (through the viral load). Unfortunately, we do not have an accurate measure of the viral load of these viruses, so cannot determine whether replication rates or loads of the viruses studied here are dramatically different. Similarly, we have no information on the replicative fidelity of the RNA-dependent RNA-polymerases of these viruses, but if there were substantial differences between their replicative accuracy that could also explain the differences observed.

The lack of diversity in ABPV and SBPV could be due to them infecting both honeybees and bumblebees. In multihost systems, species can differ in their susceptibility and response to infection (Ruiz-González et al., 2012), thus different species have very different levels of importance for the maintenance of a virus in a population. As such, a single heavily infected host species can act as sources for infection in other sympatric species. In honeybees, these viruses interact with the mite *V. destructor*, a parasite that can vector viruses and is associated with the prevalence of ABPV (Mondet et al., 2014) and SBPV (Manley et al., 2020). This vector can lead to reduced viral genetic diversity in honeybees, as shown for Deformed wing virus (Martin et al., 2012), resulting in a limited pool of virus able to spill over into other hosts. This could explain the reduction in variation we observed in the viruses known to infect honeybees relative to those only described from bumblebees. However, as this study did not contain any honeybees, we cannot rule out extensive diversity of ABPV and SBPV in honeybees, with the filtering stage being at the cross-species transmission, with only specific viral genotypes in the honeybee being able to infect bumblebees. ABPV and SBPV are both able to infect multiple genera, something which is not known to be the case for the other four viruses in this study. It is unlikely that this would lead to a lack of genetic diversity, however, as studies in divergent species have found a positive correlation (Kelley et al., 2000; Li et al., 2014) or no correlation (Zhao and Duffy, 2019) between generalism and genetic diversity.

Bumblebee-limited viruses necessarily undergo multiple independent bottlenecking events when the viruses can only survive in overwintering queens, potentially maintaining diversity through reduced selection efficiency. This would initially seem to apply to SBPV, as McMahon et al. (2015) and Manley et al. (2020) report that SBPV is often found at higher prevalences in bumblebees than in sympatric honeybees during summer. In contrast to bumblebees, over winter, honeybee colonies are maintained at population sizes in the thousands. This can maintain a level of virus in the honeybees that can then spill over into the bumblebees in the spring, which may lead to honeybee-derived SBPV variants dominating even in bumblebees. This could represent a more general effect where the fitness landscapes of viruses infecting managed species are systematically different from those infecting wild species. The hypothesized mechanism is that in a genetically homogenous, densely packed managed population, one optimal viral genotype could easily achieve dominance as the fitness landscape will be relatively constant. On the other hand, in more genetically heterogeneous wild populations with fluctuating population sizes, the fitness landscape will be less constant, and thus selection of variants on this changing fitness landscape may lead to the maintenance of more genotypes.

### Coinfection

River Luinaeg virus and Loch Morlich virus were rarely found separately in this study. They are distinct, not different segments of the same virus, because, while whole genomes are available for neither, both partial genomes include an RdRp sequence (Pascall et al., 2018). The mechanism of their transmission is unknown, but we assume that, as with most other reported bee viruses, transmission occurs at flowers.

One potential explanation for this strong association is that one of the viruses is a satellite of the other, as occurs in Chronic bee paralysis virus with Chronic bee paralysis virus satellite virus (Bailey et al., 1980). However, this seems unlikely as both virus species are observed separately, though the possibility of false negatives in the PCR reactions cannot be ruled out. Another possibility is that both viruses circulate in the population, but infection with one causes damage to the host in such a way that susceptibility to the second is dramatically increased, perhaps in a manner analogous to HIV’s synergism with TB though immune suppression (Kwan and Ernst, 2011) or influenza virus’ changing of the environment of the nasopharynx, allowing secondary bacterial invasion (Joseph et al., 2013). Viral coinfections are ubiquitously reported in prevalence studies in bees (Anderson and Gibbs, 1988; Evans, 2001; Chen et al., 2004; Nielsen et al., 2008; Bacandritsos et al., 2010; Choe et al., 2012; Mouret et al., 2013; Gajger et al., 2014; McMahon et al., 2015; Blažytė-Čereškienė et al., 2016; Thu et al., 2016; Roberts et al., 2017; Manley et al., 2020), but to our knowledge, only McMahon et al. (2015) and Manley et al. (2020) tested for a departure from random expectations of infection, and no departure was found. However, non-random associations between parasites appear common, having been reported in, among other taxa including mammals (Behnke et al., 2005; Jolles et al., 2008; Griffiths et al., 2011), birds (Clark et al., 2016), arthropods (Václav et al., 2011; Hajek and van Nouhuys, 2016) and plants (Seabloom et al., 2009; Biddle et al., 2012). Thus, while the cause is uncertain, the strength of this association makes it highly unlikely to be artefactual.

### Factors Influencing Infection

While we found potential evidence that the prevalence of River Luinaeg virus was positively associated with increased precipitation, given that only nine sites were sampled in this study, we are limited in the between-site conclusions we can draw. Despite using a regularizing prior, the risk of erroneously identifying effects can never be fully excluded in studies with small sample sizes. As such, we do not trust any conclusions drawn from the environmental variable analysis. We do believe, however, that this remains an important question. To avoid the pitfalls we have experienced, we point potential researchers to the species distribution model literature, where the question of study design for explanatory distributional studies has been extensively interrogated (Araújo et al., 2019). We recommend the descriptions of gold standard design in the supplementary materials of Araújo et al. (2019) as a starting point, despite the focus on traditional ecological rather than wildlife epidemiological questions.

## Conclusion

Here, we describe the ecological and genetic characteristics of six viruses of bumblebees. Genetic diversity is higher in the viruses we detected only in bumblebees. This could be explained if viruses in species managed for food production, such as honeybees, are less diverse than those in wild species, and outbreaks of these viruses in wild species are predominantly due to cross-species transmission from a majority to minority host. Further studies in this and other systems would be valuable to answer the question of whether there is a significant difference in diversity in viruses in managed species, those shared between managed and wild species, and those limited to wild species.

## Data Availability Statement

The datasets presented in this study can be found in online repositories. The names of the repository/repositories and accession number(s) can be found below: Sequencing data can be found at Sequence Read Archive under the following BioProject ID: PRJNA704259. All code and raw infection data is available at https://github.com/dpascall/bumble_epi_diversity.

## Author Contributions

LW and DO designed the sampling regime, acquired funding for the work, and provided the funding. LW, DO, and MT performed the sample collection. LW and DP performed the wet lab work. DP performed the data analysis and bioinformatics. DP and BC created the figures. DP wrote the first draft of the manuscript. All authors contributed to subsequent drafts and editing.

## Conflict of Interest

The authors declare that the research was conducted in the absence of any commercial or financial relationships that could be construed as a potential conflict of interest.

## References

[B1] Abascal F. Zardoya R. Telford M. J. (2010). TranslatorX: multiple alignment of nucleotide sequences guided by amino acid translations. *Nucleic Acids Res.* 38 W7–W13. 20435676 10.1093/nar/gkq291 PMC2896173

[B2] Adler L. S. Barber N. A. Biller O. M. Irwin R. E. (2020). Flowering plant composition shapes pathogen infection intensity and reproduction in bumble bee colonies. *Proc. Natl. Acad. Sci. U.S.A.* 117 11559–11565. 10.1073/pnas.2000074117 32393622 PMC7261119

[B3] Agler S. A. Burnham P. A. Bonchristiani H. F. Brody A. K. (2019). RNA virus spillover from managed honeybees (*Apis mellifera*) to wild bumblebees (*Bombus* spp.). *PLoS One* 14:e0217822. 10.1371/journal.pone.0217822 31242222 PMC6594593

[B4] Amaku M. Coutinho F. A. B. Chaib E. Massad E. (2013). The impact of Hepatitis A virus infection on Hepatitis C virus infection: a competitive exclusion hypothesis. *Bull. Math. Biol.* 75 82–93. 10.1007/s11538-012-9795-0 23192400

[B5] Anderson D. L. Gibbs A. J. (1988). Inapparent virus infections and their interactions in pupae of the honey bee (*Apis mellifera* Linnaeus) in Australia. *J. Gen. Virol.* 69 1617–1625. 10.1099/0022-1317-69-7-1617

[B6] Araújo M. B. Anderson R. P. Barbosa M. Beale C. M. Dormann C. F. Early R. (2019). Standards for distribution models in biodiversity assessments. *Sci. Adv.* 5:eaat4858. 10.1126/sciadv.aat4858 30746437 PMC6357756

[B7] Bacandritsos N. Granato A. Budge G. Papanastasiou I. Roinioti E. Caldon M. (2010). Sudden deaths and colony population decline in Greek honey bee colonies. *J. Invertebr. Pathol.* 105 335–340. 10.1016/j.jip.2010.08.004 20804765

[B8] Bailey L. Ball B. V. Carpenter J. M. Woods R. D. (1980). Small virus-like particles in honey bees associated with chronic paralysis virus and with a previously undescribed disease. *J. Gen. Virol.* 46 149–155. 10.1099/0022-1317-46-1-149

[B9] Bailey L. Gibbs A. J. (1964). Acute infection of bees with Paralysis Virus. *J. Insect Pathol.* 6 395–407.

[B10] Bailey L. Gibbs A. J. Woods R. D. (1963). Two viruses from adult honey bees (*Apis mellifera* Linnaeus). *Virology* 21 390–395. 10.1016/0042-6822(63)90200-9 14081363

[B11] Behnke J. M. Gilbert F. S. Abu-Madi M. A. Lewis J. W. (2005). Do the helminth parasites of wood mice interact? *J. Anim. Ecol.* 74 982–993. 10.1111/j.1365-2656.2005.00995.x

[B12] Biddle J. M. Linde C. Godfree R. C. (2012). Co-infection patterns and geographic distribution of a complex pathosystem targeted by pathogen-resistant plants. *Ecol. Appl.* 22 35–52. 10.1890/11-0341.1 22471074

[B13] Blažytė-Čereškienė L. Skrodenytė-Arbačiauskienė V. Radžiutė S. Čepulytė-Rakauskienė R. Apšegaitė V. Būda V. (2016). A three-year survey of honey bee viruses in Lithuania. *J. Apicult. Res.* 55 176–184. 10.1080/00218839.2016.1211389

[B14] Carpenter B. Gelman A. Hoffman M. D. Lee D. Goodrich B. Betancourt M. (2017). Stan: a probabilistic programming language. *J. Stat. Softw.* 76 1–32. 10.1017/9781108770750.002 PMC9788645 36568334

[B15] Chen Y. Zhao Y. Hammond J. Hsu H. T. Evans J. Feldlaufer M. (2004). Multiple virus infections in the honey bee and genome divergence of honey bee viruses. *J. Invertebr. Pathol.* 87 84–93. 10.1016/j.jip.2004.07.005 15579317

[B16] Choe S. E. Nguyen L. T. K. Noh J. H. Koh H. B. Jean Y. H. Kweon C. H. (2012). Prevalence and distribution of six bee viruses in Korean *Apis cerana* populations. *J. Invertebr. Pathol.* 109 330–333. 10.1016/j.jip.2012.01.003 22273697

[B17] Clark N. J. Wells K. Dimitrov D. Clegg S. M. (2016). Co-infections and environmental conditions drive the distributions of blood parasites in wild birds. *J. Anim. Ecol.* 85 1461–1470. 10.1111/1365-2656.12578 27561363

[B18] Daughenbaugh K. F. Kahnonitch I. Carey C. C. McMenamin A. J. Wiegand T. Erez T. (2021). Metatranscriptome analysis of sympatric bee species identifies bee virus variants and a new virus, Andrena-associated bee virus-1. *Viruses* 13:291. 10.3390/v13020291 33673324 PMC7917660

[B19] DePristo M. A. Banks E. Poplin R. Garimella K. V. Maguire J. R. Hartl C. (2011). A framework for variation discovery and genotyping using next-generation DNA sequencing data. *Nat. Genet.* 43 491–498. 21478889 10.1038/ng.806 PMC3083463

[B20] Durrer S. Schmid-Hempel P. (1994). Shared use of flowers leads to horizontal pathogen transmission. *Proc. R. Soc. B Biol. Sci.* 258 299–302. 10.1098/rspb.1994.0176

[B21] Evans J. D. (2001). Genetic evidence for coinfection of honey bees by acute bee paralysis and Kashmir bee viruses. *J. Invertebr. Pathol.* 78 189–193. 10.1006/jipa.2001.5066 12009798

[B22] Faurot-Daniels C. Glenny W. Daughenbaugh K. F. McMenamin A. J. Burkle L. A. Flenniken M. L. (2020). Longitudinal monitoring of honey bee colonies reveals dynamic nature of virus abundance and indicates a negative impact of Lake Sinai virus2 on colony health. *PLoS One* 15:e0237544. 32898160 10.1371/journal.pone.0237544 PMC7478651

[B23] Fick S. E. Hijmans R. J. (2017). WorldClim 2: new 1-km spatial resolution climate surfaces for global land areas. *Int. J. Climatol.* 37 4302–4315. 10.1002/joc.5086

[B24] Figueroa L. L. Grab H. Ng W. H. Myers C. R. Graystock P. McFrederick Q. S. (2020). Landscape simplification shapes pathogen prevalence in plant-pollinator networks. *Ecol. Lett.* 23 1212–1222. 32347001 10.1111/ele.13521 PMC7340580

[B25] Fürst M. A. McMahon D. P. Osborne J. L. Paxton R. J. Brown M. J. F. (2014). Disease associations between honeybees and bumblebees as a threat to wild pollinators. *Nature* 506 364–366. 10.1038/nature12977 24553241 PMC3985068

[B26] Gajger I. T. Kolodziejek J. Bakonyi T. Nowotny N. (2014). Prevalence and distribution patterns of seven different honeybee viruses in diseased colonies: a case study from Croatia. *Apidologie* 45 701–706. 10.1007/s13592-014-0287-0

[B27] Glenny W. Cavigli I. Daughenbaugh K. F. Radford R. Kegley S. E. Flenniken M. L. (2017). Honey bee (Apis mellifera) colony health and pathogen composition in migratory beekeeping operations involved in California almond pollination. *PLoS One* 12:e0182814. 10.1371/journal.pone.0182814 28817641 PMC5560708

[B28] Gordon C. H. Banyard A. C. Hussein A. Laurenson M. K. Malcolm J. R. Marino J. (2015). Canine distemper in endangered Ethiopian wolves. *Emerg. Infect. Dis.* 21 824–832. 10.3201/eid2105.141920 25898177 PMC4412237

[B29] Graystock P. Goulson D. Hughes W. O. H. (2015). Parasites in bloom: flowers aid dispersal and transmission of pollinator parasites within and between bee species. *Proc. R. Soc. B Biol. Sci.* 282 20151371. 10.1098/rspb.2015.1371 26246556 PMC4632632

[B30] Graystock P. Meeus I. Smagghe G. Goulson D. Hughes W. O. H. (2016). The effects of single and mixed infections of *Apicystis bombi* and Deformed wing virus in *Bombus terrestris*. *Parasitology* 143 358–365. 10.1017/s0031182015001614 26646676

[B31] Graystock P. Ng W. H. Parks K. Tripodi A. D. Muñiz P. A. Fersch A. A. (2020). Dominant bee species and floral abundance drive parasite temporal dynamics in plant-pollinator communities. *Nat. Ecol. Evol.* 4 1358–1367. 10.1038/s41559-020-1247-x 32690902 PMC7529964

[B32] Griffiths E. C. Pedersen A. B. Fenton A. Petchey O. L. (2011). The nature and consequences of coinfection in humans. *J. Infect.* 63 200–206. 10.1016/j.jinf.2011.06.005 21704071 PMC3430964

[B33] Hajek A. E. van Nouhuys S. (2016). Fatal diseases and parasitoids: from competition to facilitation in a shared host. *Proc. R. Soc. B Biol. Sci.* 283:20160154. 10.1098/rspb.2016.0154 27053740 PMC4843656

[B34] Horn H. S. (1966). Measurement of “Overlap” in comparative ecological studies. *Am. Nat.* 100 419–424. 10.1086/282436

[B35] Johnson P. T. J. de Roode J. C. Fenton A. (2015). Why infectious disease research needs community ecology. *Science* 349:1259504. 10.1126/science.1259504 26339035 PMC4863701

[B36] Jolles A. E. Ezenwa V. O. Etienne R. S. Turner W. C. Olff H. (2008). Interactions between macroparasites and microparasites drive infection patterns in free-ranging African buffalo. *Ecology* 89 2239–2250. 10.1890/07-0995.1 18724734

[B37] Joseph C. Togawa Y. Shindo N. (2013). Bacterial and viral infections associated with influenza. *Influenza Other Resp.* 7 105–113. 10.1111/irv.12089 24034494 PMC5909385

[B38] Joshi N. Fass J. (2011). *Sickle: A Sliding-Window, Adaptive, Quality-Based Trimming Tool for FastQ Files (Version 1.33).*

[B39] Katoh K. Standley D. M. (2013). MAFFT multiple sequence alignment software version 7: Improvements in performance and usability. *Mol. Biol. Evol.* 30 772–780. 10.1093/molbev/mst010 23329690 PMC3603318

[B40] Kelley S. T. Farrell B. D. Mitton J. B. (2000). Effects of specialization on genetic differentiation in sister species of bark beetles. *Heredity* 84 218–227. 10.1046/j.1365-2540.2000.00662.x 10762392

[B41] Kwan C. Ernst J. D. (2011). HIV and tuberculosis: a deadly human syndemic. *Clin. Microbiol. Rev.* 24 351–376. 10.1128/cmr.00042-10 21482729 PMC3122491

[B42] Lauring A. S. (2020). Within-host viral diversity: a window into viral evolution. *Ann. Rev. Virol.* 7 63–81. 10.1146/annurev-virology-010320-061642 32511081 PMC10150642

[B43] Lauring A. S. Andino R. (2010). Quasispecies theory and the behavior of RNA viruses. *PLoS Pathog.* 6:e1001005. 10.1371/journal.ppat.1001005 20661479 PMC2908548

[B44] Lee W. P. Stromberg M. P. Ward A. Stewart C. Garrison E. P. Marth G. T. (2014). MOSAIK: A hash-based algorithm for accurate next-generation sequencing short-read mapping. *PLoS One* 9:e90581. 10.1371/journal.pone.0090581 24599324 PMC3944147

[B45] Lemoine N. P. Hoffman A. Felton A. J. Baur L. Chaves F. Gray J. (2016). Underappreciated problems of low replication in ecological feld studies. *Ecology* 97 2554–2561. 10.1002/ecy.1506 27859125

[B46] Li H. Handsaker B. Wysoker A. Fennell T. Ruan J. Homer N. (2009). The sequence Alignment/Map format and SAMtools. *Bioinformatics* 25 2078–2079. 10.1093/bioinformatics/btp352 19505943 PMC2723002

[B47] Li S. Jovelin R. Yoshiga T. Tanaka R. Cutter A. D. (2014). Specialist versus generalist life histories and nucleotide diversity in Caenorhabditis nematodes. *Proc. R. Soc. B* 281:20132858. 10.1098/rspb.2013.2858 24403340 PMC3896024

[B48] Lytle C. D. Sagripanti J.-L. (2005). Predicted inactivation of viruses of relevance to biodefense by solar radiation. *J. Virol.* 79 14244–14252. 10.1128/jvi.79.22.14244-14252.2005 16254359 PMC1280232

[B49] Magoč T. Salzberg S. L. (2011). FLASH fast length adjustment of short reads to improve genome assemblies. *Bioinformatics* 27 2957–2963. 10.1093/bioinformatics/btr507 21903629 PMC3198573

[B50] Manley R. Boots M. Wilfert L. (2017). Condition-dependent virulence of slow bee paralysis virus in Bombus terrestris: are the impacts of honeybee viruses in wild pollinators underestimated? *Oecologia* 184 305–315. 10.1007/s00442-017-3851-2 28361244 PMC5487845

[B51] Manley R. Temperton B. Boots M. Wilfert L. (2020). Contrasting impacts of a novel specialist vector on multihost viral pathogen epidemiology in wild and managed bees. *Mol. Ecol.* 29 380–393. 10.1111/mec.15333 31834965 PMC7003859

[B52] Manley R. Temperton B. Doyle T. Gates D. Hedges S. Boots M. (2019). Knock-on community impacts of a novel vector: spillover of emerging DWV-B from Varroa-infested honeybees to wild bumblebees. *Ecol. Lett.* 22 1306–1315. 31190366 10.1111/ele.13323 PMC6852581

[B53] Martin S. J. Highfield A. C. Brettell L. Villalobos E. M. Budge G. E. Powell M. (2012). Global honey bee viral landscape altered by a parasitic mite. *Science* 336 1304–1306. 10.1126/science.1220941 22679096

[B54] Mbithi J. N. Springthorpe V. S. Sattar S. A. (1991). Effect of relative humidity and air temperature on survival of Hepatitis A virus on environmental surfaces. *Appl. Environ. Microb.* 57 1394–1399. 10.1128/aem.57.5.1394-1399.1991 1649579 PMC182960

[B55] McArt S. H. Koch H. Irwin R. E. Adler L. S. (2014). Arranging the bouquet of disease: floral traits and the transmission of plant and animal pathogens. *Ecol. Lett.* 17 624–636. 10.1111/ele.12257 24528408

[B56] McKenna A. Hanna M. Banks E. Sivachenko A. Cibulskis K. Kernytsky A. (2010). The genome analysis toolkit: a MapReduce framework for analyzing next-generation DNA sequencing data. *Genome Res.* 20 1297–1303. 10.1101/gr.107524.110 20644199 PMC2928508

[B57] McMahon D. P. Fürst M. A. Caspar J. Theodorou P. Brown M. J. F. Paxton R. J. (2015). A sting in the spit: widespread cross-infection of multiple RNA viruses across wild and managed bees. *J. Anim. Ecol.* 84 615–624. 10.1111/1365-2656.12345 25646973 PMC4832299

[B58] McMahon D. P. Wilfert L. Paxton R. J. Brown M. J. F. (2018). Emerging viruses in bees: from molecules to ecology. *Adv. Virus Res.* 101 251–291. 10.1016/bs.aivir.2018.02.008 29908591

[B59] Mondet F. de Miranda J. R. Kretzschmar A. Le Conte Y. Mercer A. R. (2014). On the front line: quantitative virus dynamics in honeybee (*Apis mellifera* L.) colonies along a new expansion front of the parasite *Varroa destructor*. *PLoS Pathog* 10:e1004323. 10.1371/journal.ppat.1004323 25144447 PMC4140857

[B60] Mouret C. Lambert O. Piroux M. Beaudeau F. Provost B. Benet P. (2013). Prevalence of 12 infectious agents in field colonies of 18 apiaries in western France. *Rev. Méd. Vét Toulouse* 164 577–582.

[B61] Nielsen S. L. Nicolaisen M. Kryger P. (2008). Incidence of acute bee paralysis virus, black queen cell virus, chronic bee paralysis virus, deformed wing virus, Kashmir bee virus and sacbrood virus in honey bees (*Apis mellifera*) in Denmark. *Apidologie* 39 310–314. 10.1051/apido:2008007

[B62] Oksanen J. Blanchet F. G. Kindt R. Legendre P. Minchin P. R. O’Hara R. B. (2017). *vegan: Community Ecology Package.*

[B63] Osborne J. L. Martin A. P. Carreck N. L. Swain J. L. Knight M. E. Goulson D. (2008). Bumblebee fight distances in relation to the forage landscape. *J. Anim. Ecol.* 77 406–415. 10.1111/j.1365-2656.2007.01333.x 17986207

[B64] Pascall D. J. Tinsley M. C. Obbard D. J. Wilfert L. (2018). Host evolutionary history predicts virus prevalence across bumblebee species. *bioRxiv [Preprint]* 10.1101/498717

[B65] Peat J. Goulson D. (2005). Effects of experience and weather on foraging rate and pollen versus nectar collection in the bumblebee, *Bombus terrestris*. *Behav. Ecol. Sociobiol.* 58 152–156. 10.1007/s00265-005-0916-8

[B66] R Core Development Team (2016). *R: A Language and Environment for Statistical Computing.* Vienna: R Foundation for Statistical Computing.

[B67] Roberts J. M. K. Anderson D. L. Durr P. A. (2017). Absence of deformed wing virus and Varroa destructor in Australia provides unique perspectives on honeybee viral landscapes and colony losses. *Sci. Rep.* 7:6925. 28761114 10.1038/s41598-017-07290-w PMC5537221

[B68] Ruiz-González M. X. Bryden J. Moret Y. Reber-Funk C. Schmid-Hempel P. Brown M. J. F. (2012). Dynamic transmission, host quality, and population structure in a multihost parasite of bumblebees. *Evolution* 66 3053–3066. 10.1111/j.1558-5646.2012.01655.x 23025597

[B69] Sanjuán R. Domingo-Calap P. (2019). “Genetic diversity and evolution of viral populations,” in *Reference Module in Life Sciences*, (Amsterdam: Elsevier).

[B70] Seabloom E. W. Hosseini P. R. Power A. G. Borer E. T. (2009). Diversity and composition of viral communities: coinfection of barley and cereal yellow dwarf viruses in California Grasslands. *Am. Nat.* 173 E79–E98. 19183066 10.1086/596529

[B71] Smith E. C. (2017). The not-so-infinite malleability of RNA viruses: Viral and cellular determinants of RNA virus mutation rates. *PLoS Pathog* 13:e1006254. 10.1371/journal.ppat.1006254 28448634 PMC5407569

[B72] Stan Development Team (2016). *RStan: The R Interface to Stan (R package version 2.14.1).*

[B73] Thu H. T. Thi N. Lien K. Linh M. T. Le T. H. Thi N. (2016). Prevalence of bee viruses among *Apis cerana* populations in Vietnam. *J. Apicult. Res.* 55 379–385.

[B74] Tollenaere C. Susi H. Laine A. L. (2016). Evolutionary and epidemiological implications of multiple infection in plants. *Trends Plant Sci.* 21 80–90. 10.1016/j.tplants.2015.10.014 26651920

[B75] Václav R. Ficová M. Prokop P. Betáková T. (2011). Associations between coinfection prevalence of *Borrelia lusitaniae*, *Anaplasma* sp., and *Rickettsia* sp. in hard ticks feeding on reptile hosts. *Microb. Ecol.* 61 245–253. 10.1007/s00248-010-9736-0 20711724

[B76] Watterson G. A. (1975). On the number of segregating sites in genetical models without recombination. *Theor. Popul. Biol.* 7 256–276. 10.1016/0040-5809(75)90020-9 1145509

[B77] Webster C. L. Waldron F. M. Robertson S. Crowson D. Ferrari G. Quintana J. F. (2015). The discovery, distribution, and evolution of viruses associated with *Drosophila melanogaster*. *PLoS Biol.* 13:e1002210. 10.1371/journal.pbio.1002210 26172158 PMC4501690

[B78] Whitehorn P. R. Tinsley M. C. Brown M. J. F. Darvill B. Goulson D. (2011). Genetic diversity, parasite prevalence and immunity in wild bumblebees. *Proc. R. Soc. B* 278 1195–1202. 10.1098/rspb.2010.1550 20926436 PMC3049068

[B79] Wilfert L. Long G. Schmid-Hempel P. Butlin R. Martin S. J. M. Boots M. (2016). Deformed wing virus is a recent global epidemic in honeybees driven by *Varroa mites*. *Science* 351 594–597. 10.1126/science.aac9976 26912700

[B80] Wilm A. Aw P. P. K. Bertrand D. Yeo G. H. T. Ong S. H. Wong C. H. (2012). LoFreq: a sequence-quality aware, ultra-sensitive variant caller for uncovering cell-population heterogeneity from high-throughput sequencing datasets. *Nucleic Acids Res.* 40 11189–11201. 10.1093/nar/gks918 23066108 PMC3526318

[B81] Wolf S. Moritz R. A. (2008). Foraging distance in *Bombus terrestris* L. (Hymenoptera: Apidae). *Apidologie* 39 419–427.

[B82] Wolf T. J. Ellington C. P. Begley I. S. (1999). Foraging costs in bumblebees: field conditions cause large individual differences. *Insect Soc.* 46 291–295. 10.1007/s000400050148

[B83] Wommack K. E. Nasko D. J. Chopyk J. Sakowski E. G. (2015). Counts and sequences, observations that continue to change our understanding of viruses in nature. *J. Microbiol.* 53 181–192. 10.1007/s12275-015-5068-6 25732739

[B84] Zhao L. Duffy S. (2019). Gauging genetic diversity of generalists: a test of genetic and ecological generalism with RNA virus experimental evolution. *Virus Evol.* 5:vez019. 31275611 10.1093/ve/vez019 PMC6599687

[B85] Zwart M. P. Elena S. F. (2015). Matters of size: genetic bottlenecks in virus infection and their potential impact on evolution. *Ann. Rev. Virol.* 2 161–179. 10.1146/annurev-virology-100114-055135 26958911

