# Supplementary material for: Virus Prevalence and Genetic Diversity Across a Wild Bumblebee Community
Source: Front Microbiol. 2021 Apr 22;12:650747. doi: 10.3389/fmicb.2021.650747 (PMC8100031; doi:10.3389/fmicb.2021.650747)

Supplementary Material

**Supporting Table 1** The species-site breakdown of the counts of individual bumblebees used in the study.

|  | Locations | | | | | | | | | |
| --- | --- | --- | --- | --- | --- | --- | --- | --- | --- | --- |
| Species | Dalwhinnie | Edinburgh | Glenmore | Gorebridge | Iona | Ochils | Pentlands | Staffa | Stirling | Total |
| *Bombus bohemicus* | 0 | 0 | 1 | 6 | 0 | 1 | 2 | 0 | 0 | 10 |
| *Bombus campestris* | 0 | 0 | 0 | 1 | 0 | 0 | 0 | 0 | 0 | 1 |
| *Bombus cryptarum* | 0 | 3 | 0 | 5 | 2 | 19 | 1 | 0 | 0 | 30 |
| *Bombus hortorum* | 4 | 0 | 1 | 59 | 11 | 0 | 0 | 0 | 0 | 75 |
| *Bombus jonellus* | 3 | 0 | 31 | 0 | 1 | 0 | 0 | 21 | 0 | 56 |
| *Bombus lapidarius* | 0 | 8 | 0 | 17 | 0 | 4 | 0 | 1 | 2 | 32 |
| *Bombus lucorum* | 0 | 30 | 1 | 75 | 3 | 35 | 3 | 0 | 5 | 152 |
| *Bombus magnus* | 0 | 0 | 0 | 0 | 1 | 7 | 0 | 0 | 0 | 8 |
| *Bombus monticola* | 0 | 0 | 2 | 0 | 0 | 4 | 3 | 3 | 0 | 12 |
| *Bombus pascuorum* | 0 | 43 | 0 | 47 | 3 | 44 | 2 | 1 | 14 | 154 |
| *Bombus pratorum* | 0 | 29 | 0 | 13 | 0 | 1 | 0 | 0 | 3 | 46 |
| *Bombus sylvestris* | 0 | 0 | 0 | 1 | 0 | 1 | 1 | 0 | 0 | 3 |
| *Bombus terrestris* | 0 | 50 | 0 | 104 | 0 | 12 | 1 | 0 | 13 | 180 |
| Total | 7 | 163 | 36 | 328 | 21 | 128 | 13 | 26 | 37 | 759 |

**Supporting Table 2** The PCR primers for each virus used in the study

| **Virus** | **Primer F** | **Primer R** | **Ref** |
| --- | --- | --- | --- |
| Loch Morlich virus | AGTGGTGGAGATGGAGACGA | CCACAGATACCAGTGGCGTA | Pascall et al. 2018 |
| River Luineag virus | ACCAGGTGGAACTCGTGTTT | GTACTCTGGACCTTTGCCGT | Pascall et al. 2018 |
| Mayfield virus 1 | TATCCGCCGGCGTAATCTTC | GGATCTGATCCGTAGCGTGG | Pascall et al. 2018 |
| Mayfield virus 2 | CGGCTGCGTTGCGTAGTATA | ACCTGCCGTGCTAACAAATA | Pascall et al. 2018 |
| Slow bee paralysis virus | GAGATGGATMGRCCTGAAGG | CATGAGCCCAKGARTGTGAA | Lena Wilfert (*pers comm)* |
| Acute bee paralysis virus | CYATGGACACACCCTATGTG | CGCCATTTTGCTACTTCTCC | Lena Wilfert (pers comm) |

**Supporting Table 3** Approximations to Watterson’s estimator for sites over a homologous genomic region within the RdRp gene by host species. Combinations that weren’t tested due to low numbers of mapping reads are marked with “-“. Entries with 0.000 had no observed variation over the region under study. The point estimate is at the median predicted number of infected individuals and uncertainty corresponds to the values at the 5^th^ and 95^th^ percentile of the predicted number of infected individuals (see methods).

|  | Mixed *Bombus* pool | *Bombus terrestris* pool | *Bombus lucorum* pool | *Bombus pascuorum* pool |
| --- | --- | --- | --- | --- |
| River Luinaeg virus | 0.029  (0.029-0.030) | - | - | - |
| Loch Morlich virus | 0.026  (0.025-0.027) | - | - | - |
| Mayfield virus 1 | 0.010  (0.009-0.010) | 0.015  (0.014-0.015) | 0.024  (0.023-0.025) | - |
| Mayfield virus 2 | 0.035  (0.034-0.036) | 0.014  (0.012-0.016) | - | 0.029  (0.028-0.030) |
| Acute bee paralysis virus | 0.000  (0.000-0.000) | - | 0.000  (0.000-0.000) | 0.000  (0.000-0.000) |
| Slow bee paralysis virus | 0.008  (0.008-0.008) | 0.002  (0.002-0.002) | 0.003  (0.003-0.003) | 0.006  (0.006-0.006) |

**Supporting Figure 1** The median read depth over called variants for each pool for each virus plotted against the approximation to Watterson’s estimator. Errors correspond to estimation at the end points 90% credible interval for the number of extra untested positives from the pools (see methods). Combinations were excluded if the median read depth over called differences from the consensus was less than 20. Colours correspond to viruses and shapes to the pools.

**
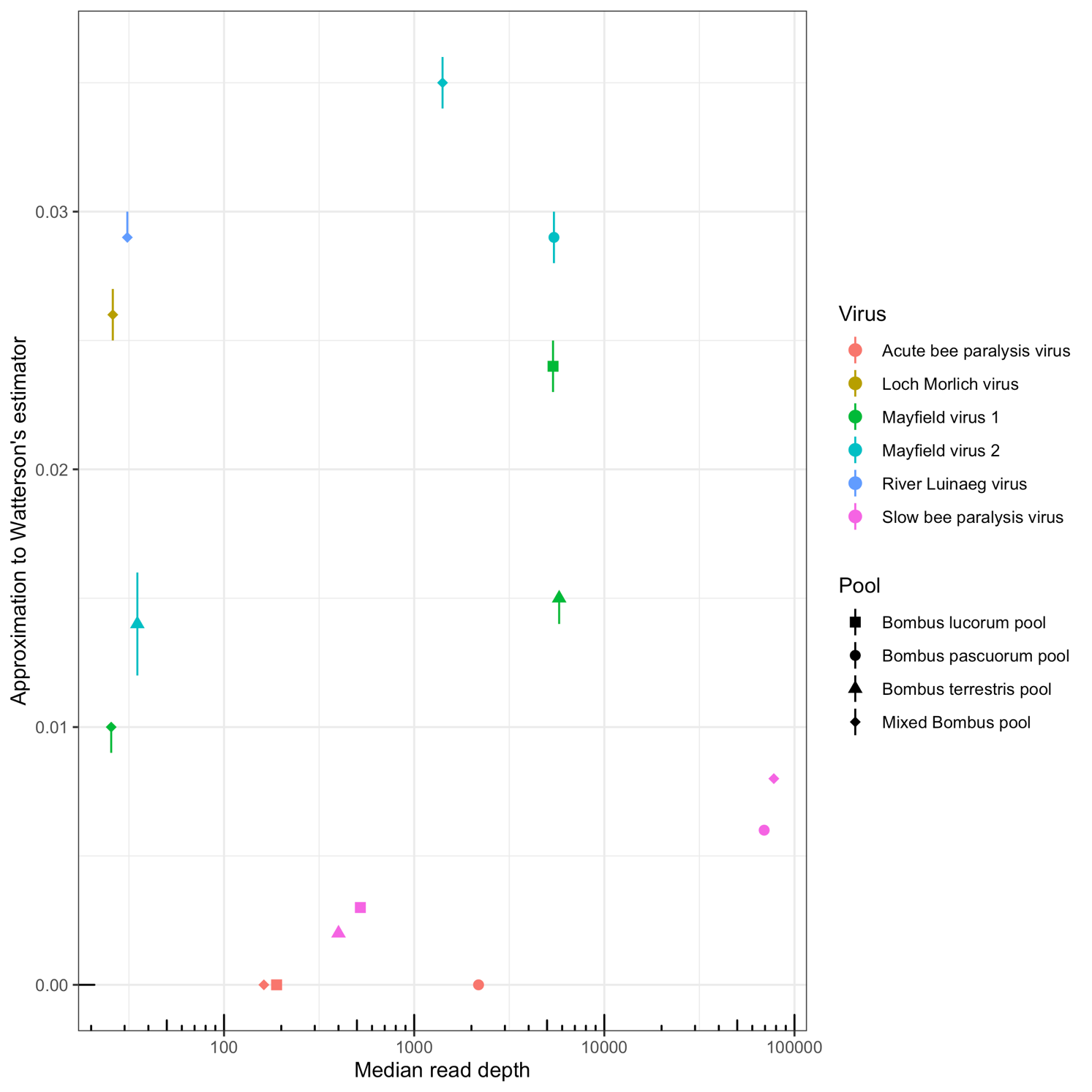
**

**Supporting Figure 2** The prevalence of Acute bee paralysis virus, Loch Morlich virus, Mayfield virus 1, Mayfield virus 2, River Luinaeg virus and Slow bee paralysis virus in each sampled host species in each site. The point estimate is the posterior mode, with 50% shortest posterior intervals represented by the thick lines and 90% shortest posterior intervals represented by the thin lines. Untested combinations are left blank. Species are coloured by their corresponding colour in Figure 1 for ease of reading.


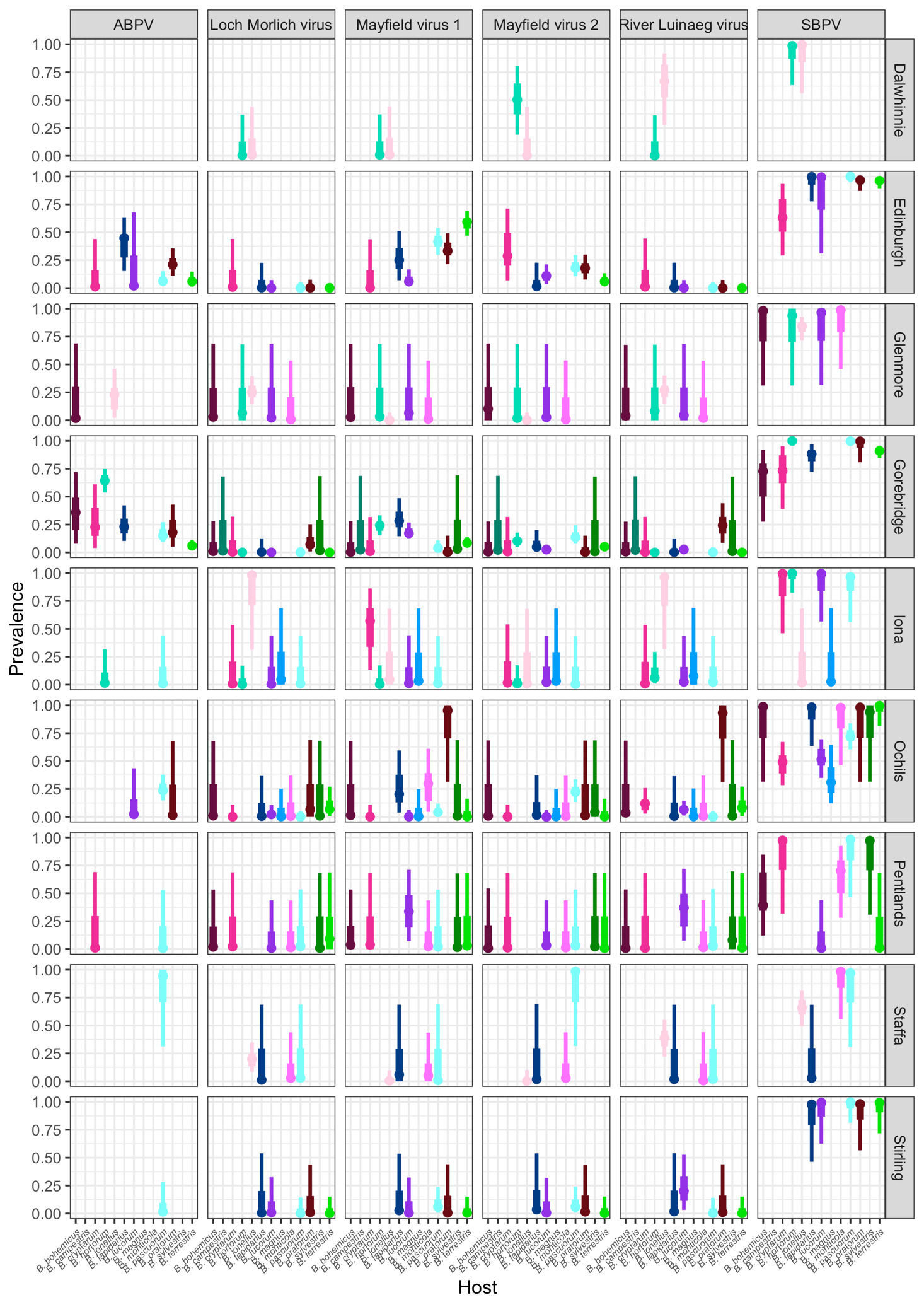


**Supporting Figure 3** The estimates for each parameter in each virus from the multivariate probit model. The estimate is the posterior mode, with 50% shortest posterior intervals represented by the thick lines and 90% shortest posterior intervals represented by the thin lines.


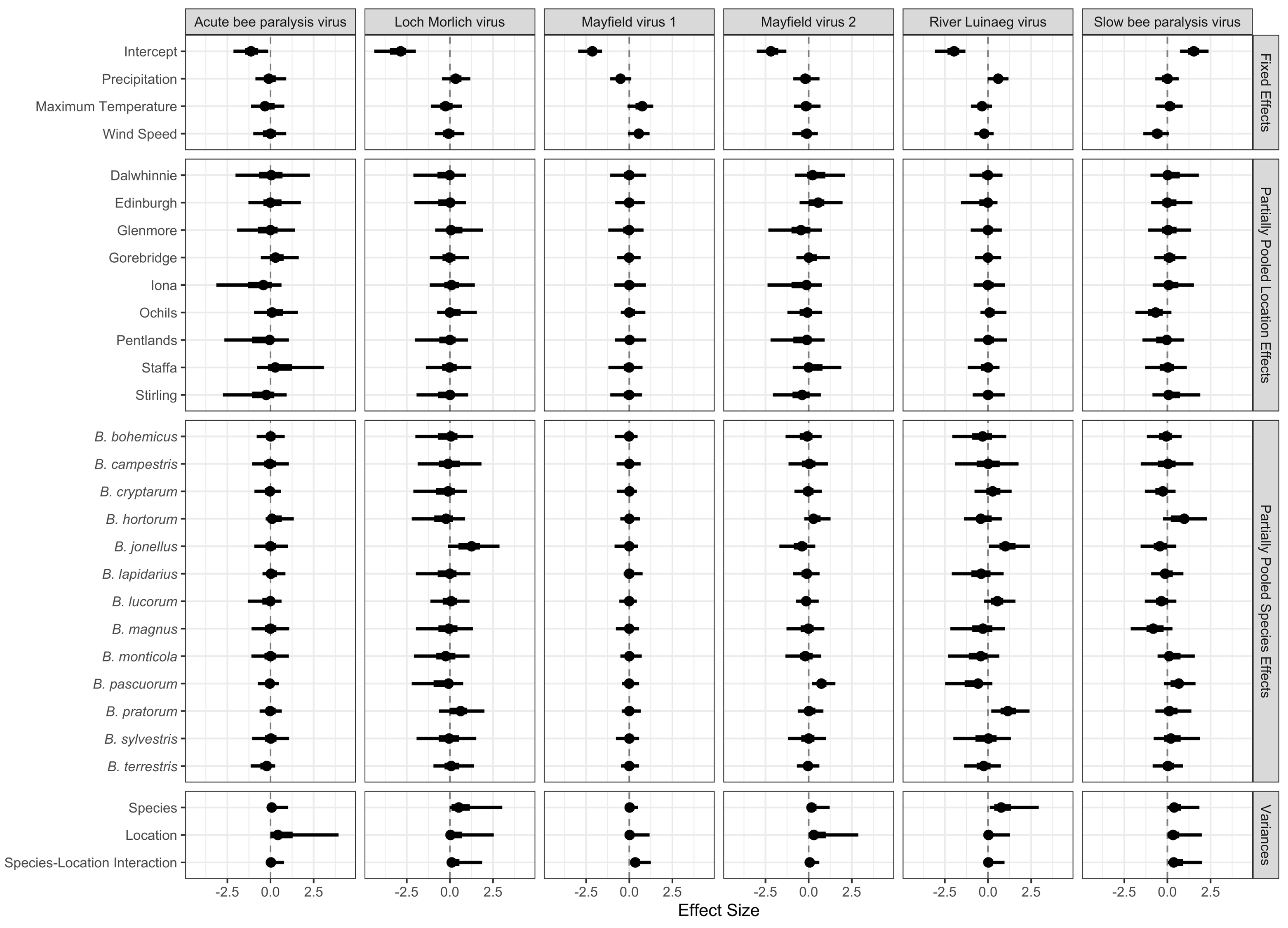

Supplement: Supplementary file 1 [file Data_Sheet_1.docx]
